# Highly sensitive troponin t (hstnt) profile for outcome prediction after cardiac surgery

**DOI:** 10.1186/2197-425X-3-S1-A108

**Published:** 2015-10-01

**Authors:** AS Omar, S Sudarsanan, S Hanoura, H Osman, P Sivadasan, Y Shouman, AK Tuli, R Singh, A Al Khulaifi

**Affiliations:** Hamad Medical Corporation, Doha, Qatar; Faculty of Medicine, Critical Care Medicine, Beni_Suef, Egypt; Hamad Medical Corporation, Cardiothoracic Surgery-Heart Hospital, Doha, Qatar; Hamad Medical Corporation, Medical Research Center, Biomedical Statistics, Doha, Qatar

## Introduction

Changes in cardiac mediators remain a subject of research interest. Instantly obtainable biomarkers that are performed routinely, are inexpensive and are characterized by linkages to outcome in cardiac surgery settings are optimum. Post-operative conventional cardiac troponins are linked to short- and middle-length outcomes [1] but highly sensitive troponin T (hsTnT) has not been extensively evaluated in the same settings.

## Objectives

To assess the ability of hsTnT to prognosticate outcomes in cardiac surgery.

## Methods

We conducted a single-center, prospective observational study over 2 years. We analyzed the data from all patients who underwent cardiac surgery. We recruited 413 patients with a mean age of 54.9 ± 10.9 years. The patients were divided into two groups based on hsTnT level, which is analogous to creatine kinase MB (CK-MB) and indicates myocardial injury (with and without myocardial infarction) [2]. The receiver operator curve (ROC) was used to determine this relation, retrieving a level of 2309 ng/L and showing an 80% sensitivity and an 86% specificity (figure). We used a *t*-test to compare variables and multivariate analysis was conducted for significant variables.

**Figure 1:**
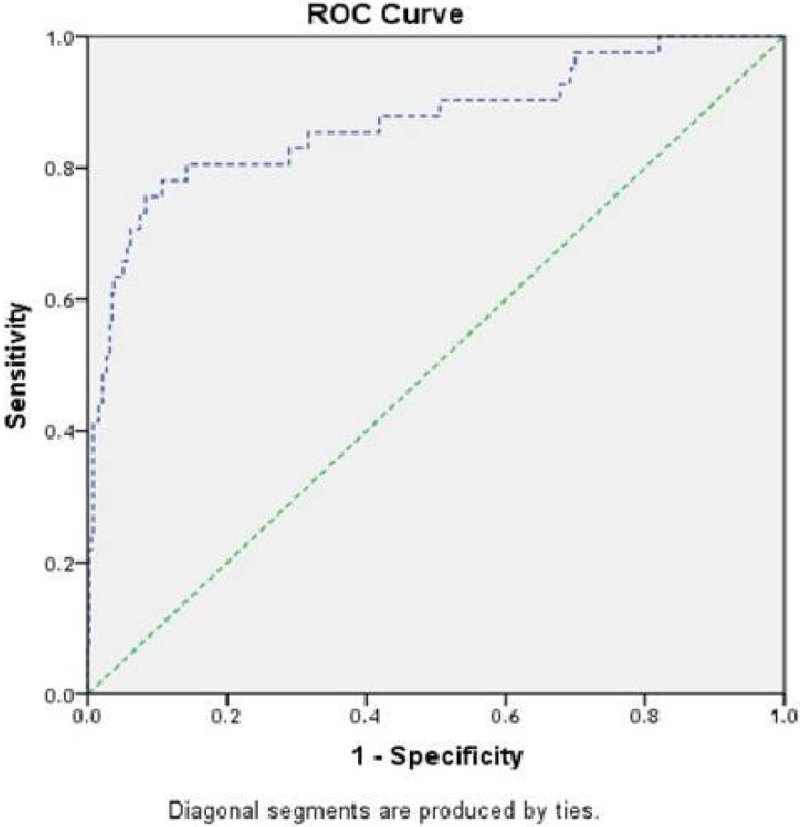
**ROC for hsTnT associated with a high CK-MB.**

## Results

Two groups (group A (372 patients) and group B (41 patients)) were both matched for age, body mass index, diabetes mellitus association, serum creatinine, Euroscore, aortic cross clamp and cardiopulmonary bypass time, and total length of anesthesia. Patients with hsTnT levels of 2309 ng/L or lower had a better outcome in terms of inotropes need, lengths of ventilation (LOV), ICU and hospital stay, and post-operative complications. Multivariate analysis revealed significant relations of the given level with operative emergency (p = 0.001); the level was a predictor for a longer duration of mechanical ventilation (p = 0.01) and post-operative atrial fibrillation (POAF) (p = 0.003) (Table 1). Moreover, 9 patients (21.9%) in group B had perioperative myocardial infarctions.

**Table 1:** Multivariate logistic regression for hsTnT.

Variable	Adjusted OR	95% CI	P-Value
Operation emergency	10.21	2.5-41.3	0.001
LOV	1.01	1.00-1.02	0.01
AKI	0.84	0.32-2.20	0.72
POAF	4.79	1.7-13.5	0.003
Mortality	3.71	0.4-32.9	0.24

CI: confidence interval; LOV: length of ventilation; AKI: acute kidney injury; POAF: post-operative atrial fibrillation

## Conclusions

Outcome prediction in cardiac surgery in terms of PMI, LOV and POAF could be enhanced by using a set level of hsTnT; this profile serves as a powerful laboratory marker to identify high-risk patients.

## Grant Acknowledgment

We thank all members of cardiothoracic surgery department and the medical research center of Hamad medical corporation.
